# Supplementary material for: Encapsulation in a Bacterial Microcompartment Shell Improves Thermal Stability of a Glycolytic Enzyme
Source: ACS Synth Biol. 2026 May 4;15(5):1980–92. doi: 10.1021/acssynbio.6c00074 (PMC13185154; doi:10.1021/acssynbio.6c00074)
Supplement: Supplementary file 1 [file sb6c00074_si_001.pdf]

Supporting Information for Publication

Encapsulation in a bacterial microcompartment shell improves thermal stability of a glycolytic enzyme

Nicholas M. Tefft[1], Neetu S. Yadav[1], Megan C. Gruenberg Cross[1], Charles D. Swiggett[1], Kristin N. Parent[1], Josh V. Vermaas[1] and Michaela A. TerAvest[1]*

[1]Department of Biochemistry and Molecular Biology, Michigan State University, East Lansing, MI, USA, 48824

*Corresponding author: teraves2@msu.edu

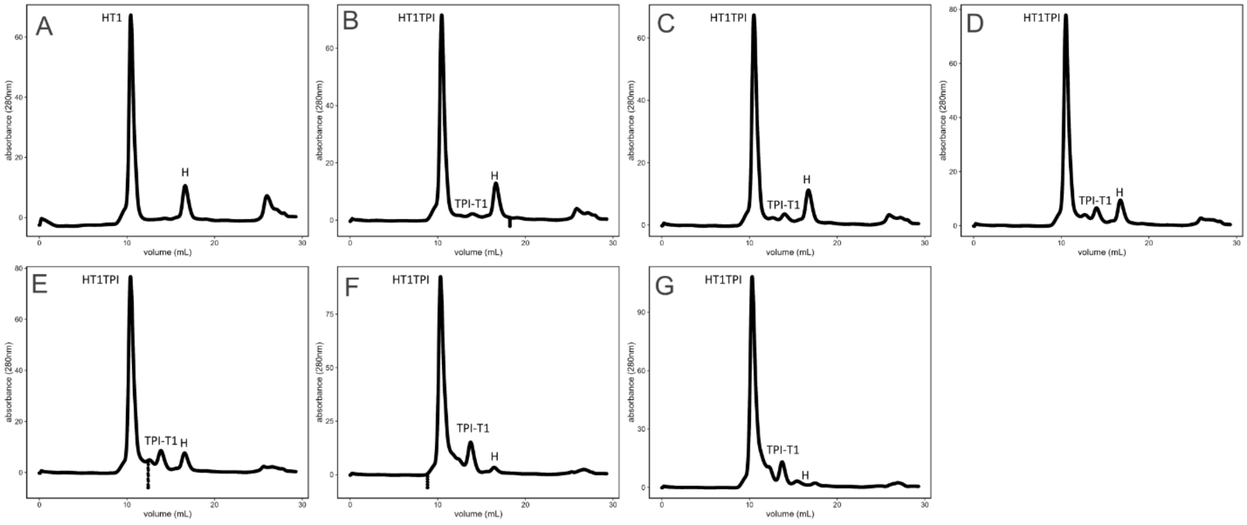

Figure S1. Size exclusion chromatograms of variably loaded HT1 shells. A. HT1 **B.** HT1 TPI 6:1 **C.** HT1 TPI 3:1 **D.** HT1 TPI 3:2 **E.** HT1 TPI 1:1 **F.** HT1 TPI 1:2 **G.** HT1 TPI 1:3

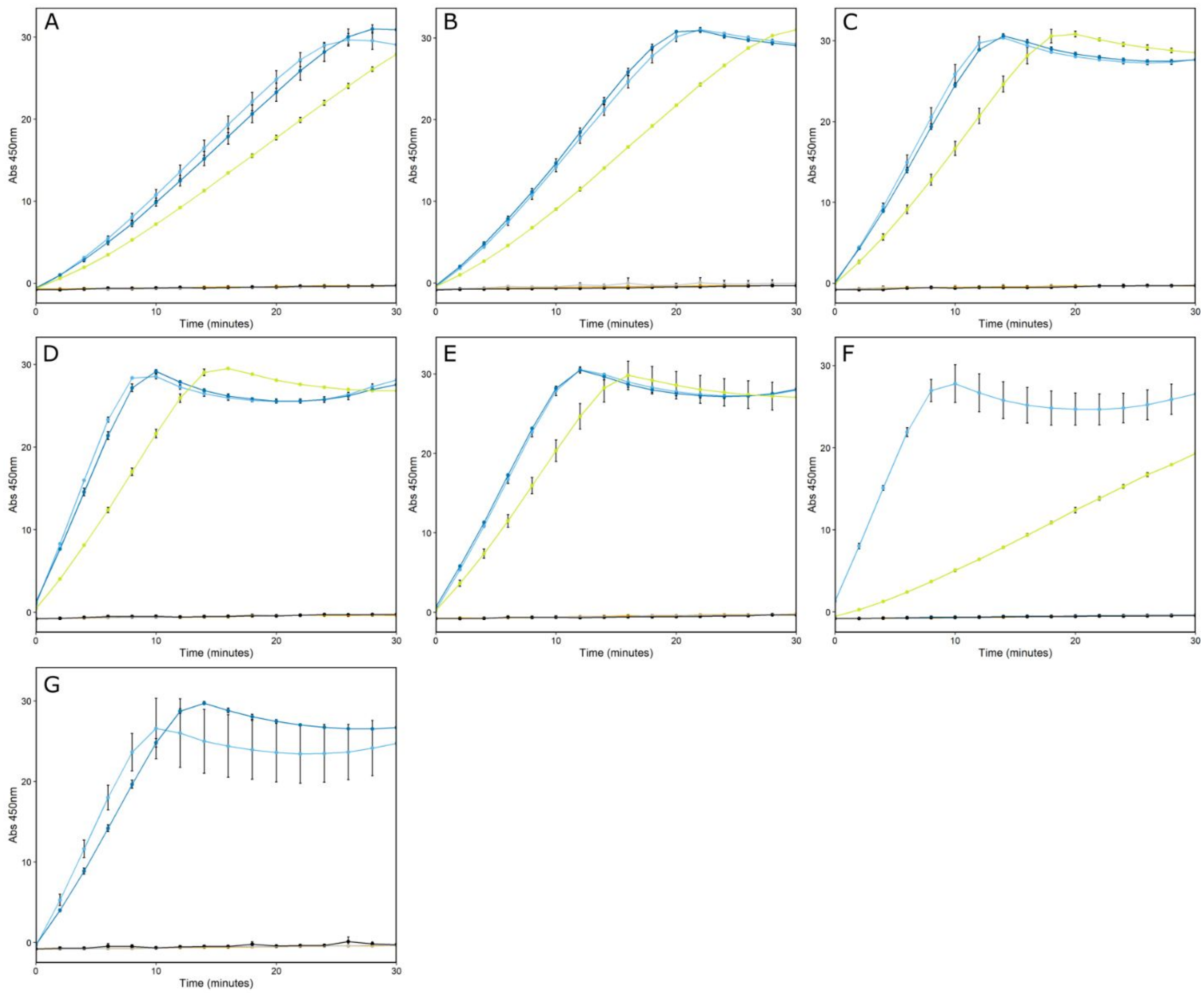

Figure S2. TPI activity of heat-treated HO shells. A. HT1 TPI 6:1 **B.** HT1 TPI 3:1 **C.** HT1 TPI 3:2 **D.** HT1 TPI 1:1 **E.** HT1 TPI 1:2 **F.** HT1 TPI 1:3 **G.** TPI. Light blue is 37°C, dark blue is 47°C, yellow is 57°C, orange is 67°C, grey is 77°C, black is 87°C.

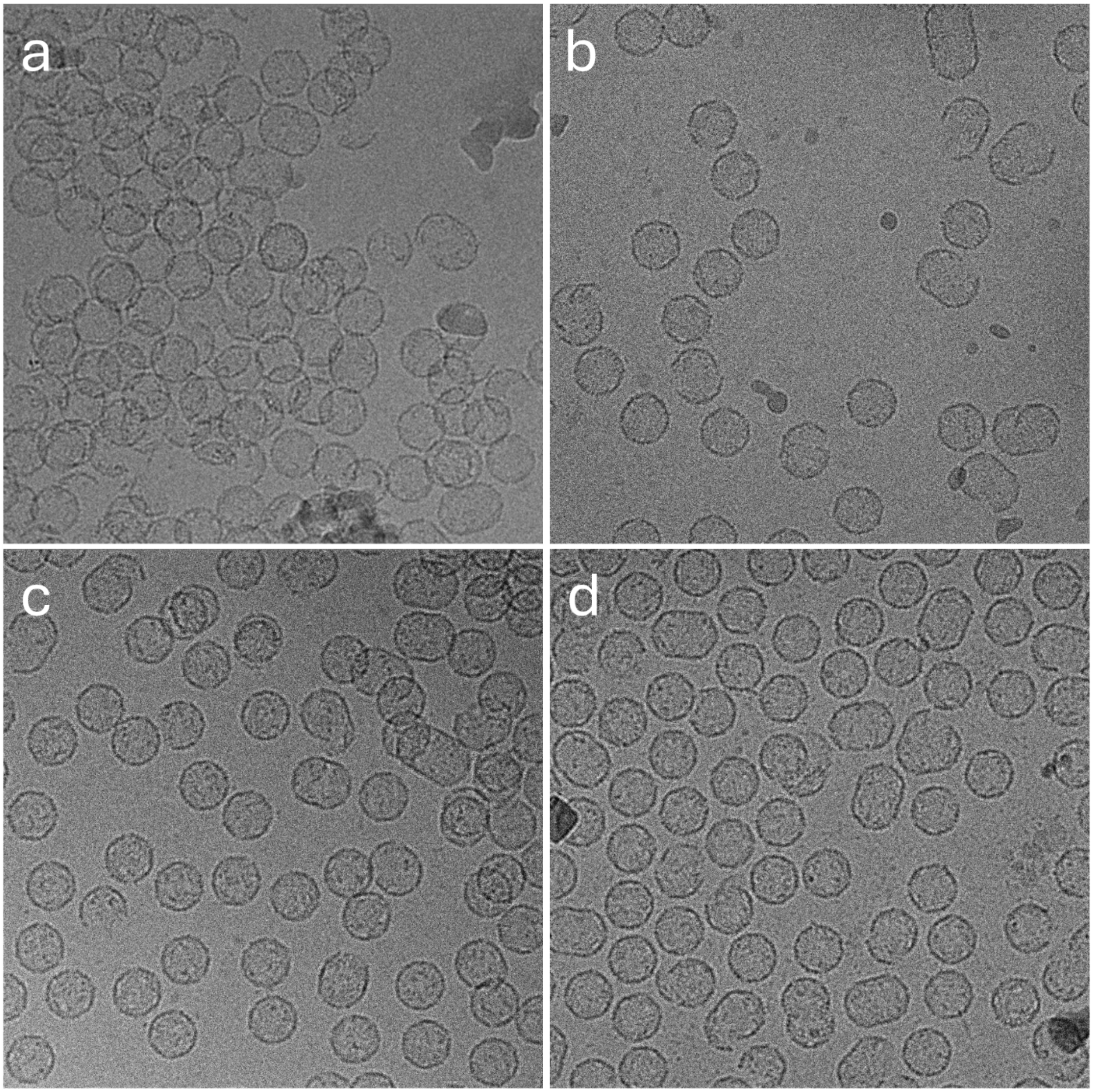

Figure S3. Cyro EM images of heat treated TPI HO-shells. Shells and cargo remain intact after heating to 55˚C. **A.** HT1 untreated **B.** HT1 heat treated **C.** HT1 TPI 1:1 untreated **D.** HT1 TPI 1:1heat treated.

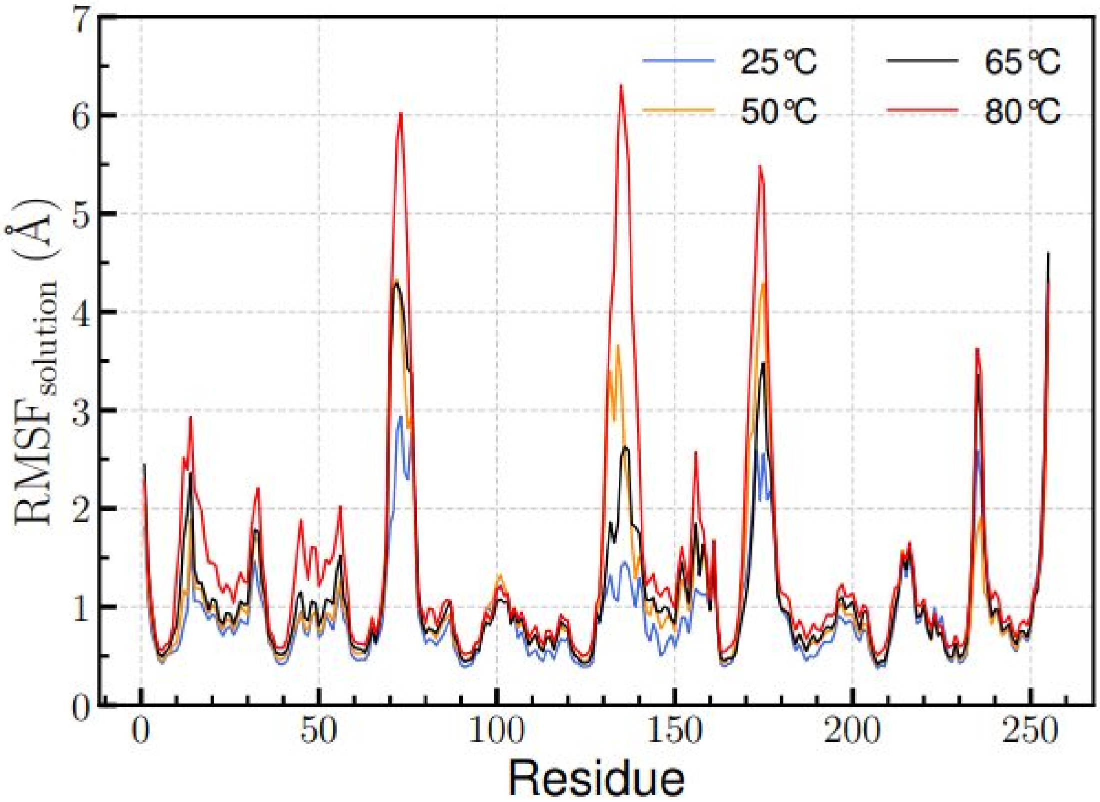

Figure S4. RMSF of TPI in aqueous system.

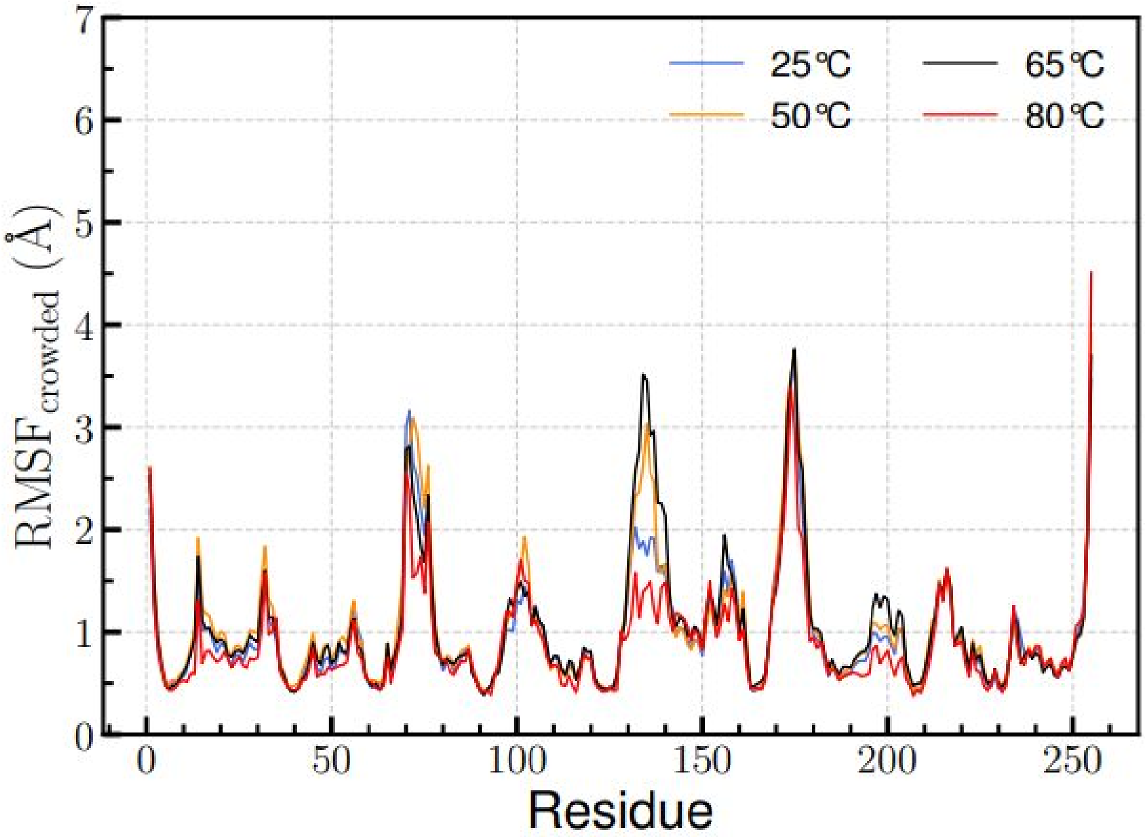

Figure S5. RMSF of TPI in crowded system.

Table S1. Dynamic Light Scattering analysis of shell samples. Each shell average diameter is an average of 3 reads.

Shell	average diameter (nm)	stdev
HT1	44.22	0.57
HT1 TPI 6:1	46.05	0.38
HT1 TPI 3:1	45.96	0.23
HT1 TPI 3:2	46.65	0.22
HT1 TPI 1:1	47.29	0.11
HT1 TPI 1:2	48.81	0.21

HT1 TPI 1:3	57.98	1.00
